# Antioxidant Activity of Lignin Phenolic Compounds Extracted from Kraft and Sulphite Black Liquors 

**DOI:** 10.3390/molecules15129308

**Published:** 2010-12-16

**Authors:** Hélio Faustino, Nuno Gil, Cecília Baptista, Ana Paula Duarte

**Affiliations:** 1 Textile & Paper Materials Research Unit, University of Beira Interior, 6201-001 Covilhã, Portugal; 2 Department of Chemical and Environmental Engineering, ESTT, Polytechnic Institute of Tomar, 2300-313 Tomar, Portugal; E-Mail: cecilia@ipt.pt (C.B.); 3 Faculty of Health Sciences, University of Beira Interior, 6201-001 Covilhã, Portugal; 4 Faculty of Health Sciences, Health Sciences Research Centre, 6201-001 Covilhã, Portugal

**Keywords:** *Eucalyptus globulus*, antioxidant activity, black liquors, DPPH method, total phenolic content

## Abstract

The antioxidant activity of the phenolic compounds present in industrial black liquors obtained from the two cooking processes (kraft and sulphite) used in Portugal to produce *Eucalyptus globulus* pulp was evaluated. The black liquors treated at several pH values were extracted with ethyl acetate. Phenolic fractions were further separated by liquid chromatography of the crude extracts of kraft liquor at pH = 6 and sulphite liquor at the original pH. Total phenolic content was determined in terms of gallic acid equivalents (Folin-Ciocalteu colorimetric method), and the antioxidant activity in the crude extracts at several pH values and in the separated fractions was measured using the DPPH test for radical scavenging capacity. The total phenolic content of crude extracts and separated fractions ranged from 92.7 to 181.6 and from 91.6 to 1,099.6 mg GAE/g, respectively, while the antioxidant activity index (AAI) ranged from 2.20 to 3.41 and from 2.21 to 11.47 respectively, showing very strong antioxidant activity in all studied cases. The fractions separated by column chromatography were submitted to mass spectrometry analysis and the results were compared to others in the literature of natural products, mainly from Eucalyptus, and the characteristic bands of functional groups were identified by ^1^H-NMR and FTIR. These methods allowed the identification of 17 phenolic compounds.

## 1. Introduction

Chemical pulping refers to the most often used processes to produce paper pulp and is based on the principle of lignin dissolution, enabling the liberation of fibers from the wood matrix. The fibers are then used in paper production after being submitted to several chemical and physical operations. Wood delignification can be performed under alkaline, neutral and acidic conditions, involving different mechanisms and outcomes. Kraft pulping is nowadays the most important chemical pulping process. In a kraft cook, wood is delignified by the action of a strong alkaline solution (pH~14) composed mainly by sodium hydroxide and sodium sulphide, at a temperature of 160-170 ºC. The reactions occurred during pulping promote the cleavage of the lignin macromolecule, the solubilisation of its degraded small fragments (that remain in the final solution named black liquor) and the liberation of cellulosic fibers (unbleached kraft pulp). The sulphite process is an acidic delignification process, whose cooking liquor (pH~5) is a mixture of free sulphurous acid and combined sulphurous acid in the form of bisulphite ion. The objective of its action is also lignin depolymerisation, in that case, by sulfonation and hydrolysis. Black liquors from the cooking process in kraft and sulphite pulp mills contain chemicals and dissolved wood substances. About half of the wood components is dissolved into the black liquor. The dissolved organic compounds consist mainly in degraded lignin and also hemicelluloses and cellulose degradation products. The liquor is normally incinerated with recovery of inorganic cooking chemicals and production of steam; in fact, it has been estimated that only 1–2% of lignin is isolated from pulping liquors and used for specialty products [1]. Valuable chemical properties and functions of lignin and hemicelluloses thus wasted when the black liquor is simply burnt at the mill site for energy recovery [2]. 

Phenols derived from biomass oils are valuable and useful chemicals. They have some pharmacological properties and also could have an inhibitor effect on the expression of HIV-1 gene [3]. They can also be used for the production of adhesives and for the synthesis of polymers [4,5]. 

Natural antioxidants may be used to preserve food from lipid peroxidation and oxidative damage occurring in living systems. Their action in oil rancidity retardation or inhibition is also noticed and assumes an industrial important role. Antioxidants can also prevent the loss of food colour, flavour and active vitamins content, providing the stabilization of the molecules involved in such characteristics. The synthetic antioxidants include butylated hydroxyanisole and butylated hidroxytoluene (BHA and BHT, respectively), propyl gallate (PG) and tertbutylhydroquinone (TBHQ). Their manufacture costs, the relative poor efficiency of natural tocopherols (also used as antioxidant agents) and the need of increased food additive safety gave rise to a crescent demand on other natural and safe antioxidants sources. The search for cheap and widespread feedstocks for this purpose has led to the evaluation of residual materials, including several leaves, seeds and peels, generally considered as wastes [6,7]. The fibrous part of vegetal biomass can yield antioxidants after hydrolytic processing [8,9].

Despite the fact that the utilization of crude extracts as antioxidants instead of pure compounds or purified fractions is a more favorable alternative from an economic point of view and in some cases crude extracts have proved to be superior to synthetic mixtures of main components [8], the separation of compounds present in the crude extract can eliminate eventual undesirable chemicals and help in the identification of the most important phenolic compounds present. Several techniques for separation of phenolic compounds from biomass have been reported, like electrophoretic separation [10,11], reversed-phase liquid chromatography [12], thin-layer chromatography [5], liquid chromatography [13,14], two-dimensional liquid chromatography [15], on-chip micellar electrokinetic chromatography [16], high-performance liquid chromatography [17,18] or distillation [19]. 

In the present study black and sulphite liquors were extracted with ethyl acetate, which has proven to be a good solvent for the extraction of phenolic fractions and can be easily recovered by vacuum evaporation from the obtained crude extracts [20]. The crude extracts were subjected to liquid column chromatography using 10%chloroform/ethyl acetate as eluent. Both crude and separated fractions were analyzed in terms of gallic acid equivalents by the Folin-Ciocalteu method [21]; respective antioxidant activity index (AAI) were determined by the 2,2-diphenyl-1-picrylhydrazyl method proposed by Scherer and Godoy [22]. The separated fractions were also characterized by ^1^H-NMR, mass and infrared spectroscopy. 

## 2. Results and Discussion

### 2.1. Influence of pH in total phenolic content and antioxidant activity index of the crude black liquor extracts

**Figure 1:**
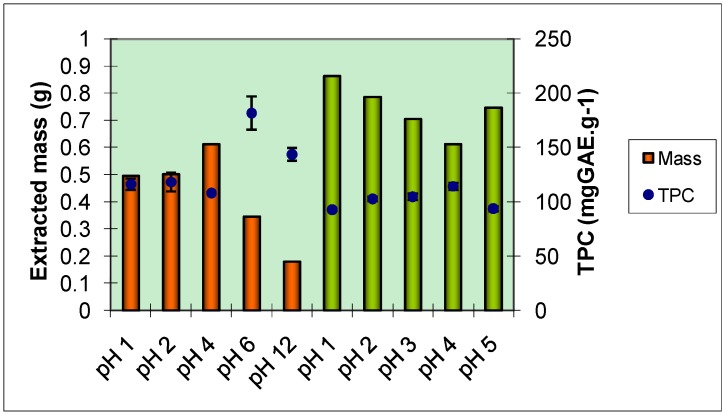
Total phenolic content of black liquors crude extracts at several pH: orange columns – kraft; green columns – sulphite.

The spectrophotometric results obtained from the Folin-Ciocalteu method concerning the total phenolic content (TPC) of the crude extracts of kraft black liquor and the crude extracts of sulphite black liquor are given in Figure 1.

Considering that both delignification methods give rise to polysaccharide depolymerisation and that some sugars can remain after the ethyl acetate extractions performed and consequently interfere with the determination by reducing the Folin-Ciocalteu reaction mixture, these figures only intend to provide a relative number for comparison between the two cooking processes. Under these conditions this method can’t determine the absolute value of phenolic content, nevertheless it is still widely used for total phenolic quantitative purposes [21,23]. 

The highest extraction yield for total phenolic compounds was obtained when kraft black liquor was extracted at pH = 6, which gave a value of 181.6 ± 15.2 mgGAE.g^-1^. In the other series of extracts, the extract at pH = 4 showed the greater TPC, 114.2 ± 3.2 mgGAE.g^-1^. It appears that the pH value has no linear effect on the extracted mass and on the total phenolic content of the crude extracts. Table 1 displays the antioxidant activity results determined by the DPPH (2,2-diphenyl-1-picrylhydrazyl radical) method for all the extracted samples obtained from both type of black liquors, using three different DPPH concentrations, namely 31.6, 49.0 and 78.8 mg·mL^-1^. 

**Table 1:** Values of the antioxidant activity index (AAI) with different final concentrations of DPPH for the crude extracts from kraft and sulphite black liquors at different pH.

Kraft black liquor					Sulphite black liquor				
pH	DPPH conc. (mg.mL^-1^)	Mean IC_50_	Mean AAI ^(a)^	Total Mean AAI ^(a)^	pH	DPPH conc. (mg.mL^-1^)	Mean IC_50_	Mean AAI ^(a)^	Total Mean AAI ^(a)^
1	31.6	21.51	2.17 ± 0.03	2.20 ± 0.18	1	31.6	12.82	2.54 ± 0.02	2. 92 ± 0.34
	49.0	29.15	2.16 ± 0.25			49.0	18.31	2.88 ± 0.02	
	78.8	34.03	2.27 ± 0.23			78.8	26.99	3.32 ± 0.06	
2	31.6	22.03	2.10 ± 0.12	2.48 ± 0.30	2	31.6	11.36	2.91 ± 0.03	3.29 ± 0.35
	49.0	24.02	2.57 ± 0.02			49.0	16.35	3.26 ± 0.01	
	78.8	30.92	2.76 ± 0.02			78.8	24.38	3.71 ± 0.02	
4	31.6	19.64	2.02 ± 0.08	2.31 ± 0.25	3	31.6	12.36	2.67 ± 0.03	2.98 ± 0.31
	49.0	22.84	2.35 ± 0.01			49.0	18.67	2.89 ± 0.04	
	78.8	30.04	2.58 ± 0.07			78.8	27.41	3.37 ± 0.06	
6	31.6	13.19	3.00 ± 0.01	3.41 ± 0.33	4	31.6	9.33	2.88 ± 0.06	3.12 ± 0.28
	49.0	15.16	3.47 ± 0.02			49.0	15.65	3.01 ± 0.02	
	78.8	20.80	3.76 ± 0.04			78.8	21.95	3.48 ± 0.01	
12	31.6	10.44	2.61 ± 0.03	2.74 ± 0.20	5	31.6	10.88	2.50 ± 0.06	2.60 ± 0.34
	49.0	18.67	2.62 ± 0.03			49.0	20.59	2.34 ± 0.36	
	78.8	26.07	3.01 ± 0.05			78.8	25.99	2.96 ± 0.11	

(a) - value displayed with the standard deviation.

In the assay of kraft extracts, the pH value that provides the most important antioxidant activity index (AAI) was also pH 6 (3.41 ± 0.33), despite the fact that, at this pH value, the extracted mass was one of the lowest (Figure 1), the TPC and AAI exhibited were the highest. For this reason, the separation of fractions from this liquor was made with the extracts obtained at pH = 6.

In the sulphite extracts series, the extract at pH = 2 exhibited the greater AAI value (3.29 ± 0.35). The separation of fractions from the sulphite liquor was made from the crude extract obtained at the original pH (pH = 5), as no treatment would be preferable from an industrial point of view.

As occurred before with TPC, it seems that there is no linear dependence between the antioxidant activity and the extraction pH value. According to the scale proposed by Scherer and Godoy [22], namely, poor antioxidant activity when AAI < 0.5, moderate antioxidant activity when 0.5 < AAI < 1.0, strong antioxidant activity when 1.0 < AAI < 2.0 and very strong antioxidant activity when AAI > 2.0, one can observe that all extracts showed very high antioxidant activity. 

**Figure 2:**
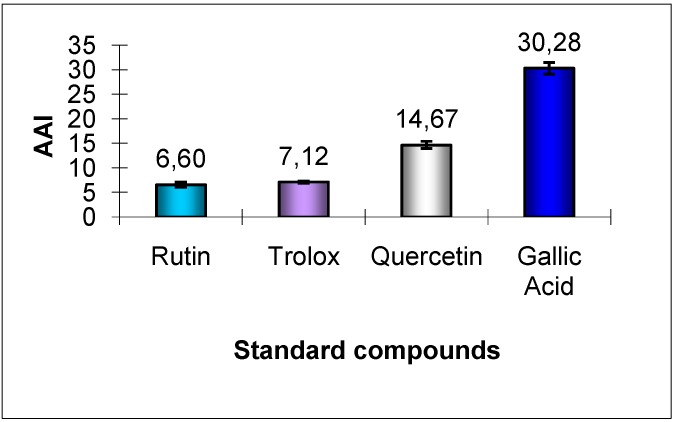
Antioxidant activity indexes of the standard compounds.

The phenolic compounds used as standards were rutin, trolox, quercetin and gallic acid, that exhibited the antioxidant activity indexes (AAI) displayed in Figure 2.

**Figure 3:**
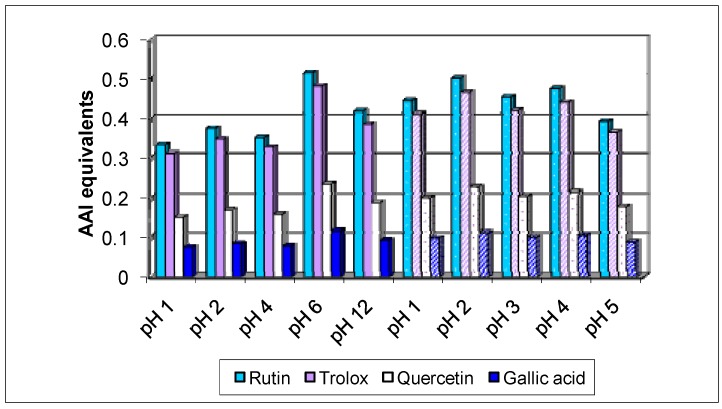
AAI equivalents of the black liquors crude extracts in comparison with several standards: columns with solid color – kraft liquor; columns with patterns – sulphite liquor.

Figure 3 presents the AAI equivalents for the extracts from kraft and sulphite black liquors, in comparison with the phenolic standards used.

From these graphics it could be seen that the crude extracts from sulphite black liquor present higher antioxidant activities that the ones from kraft black liquor, which could be related with the corresponding higher TPC content. 

### 2.2. Antioxidant activity index for the separated fractions

It was possible to separate six fractions (K1 to K6) from the kraft black liquor selected crude extract. The fractions K1 and K4 had no solubility in methanol, unlike the normal behaviour of phenols, so the TPC and AAI for these fractions were not determined. From the chosen sulphite crude four fractions (S1 to S4) were separated. Table 2 shows the Rf, the mass and TPC of the separated fractions obtained from the kraft and sulphite chosen crude extracts.

**Table 2:** Characteristics of separated fractions from the kraft and sulphite black liquors.

Kraft black liquor pH = 6				Sulphite black liquor pH = 5			
Fraction	Rf ^(a)^	Mass of the fraction (g)	TPC ^(b)^ (mgGAE.g-1)	Fraction	Rf ^(a)^	Mass of the fraction (g)	TPC ^(b)^ (mgGAE.g-1)
K2	0.61	0.0492	293.5 ± 8.71	S1	0.76	0.4897	363.1 ± 11.13
K3	0.38	0.1852	146.9 ± 15.94	S2	0.51	0.1654	612.4 ± 22.64
K5	0.20	0.2487	198.1 ± 2.08	S3	0.32	0.2363	1099.6 ± 2.48
K6	0.05	0.1529	91.6 ± 0.17	S4	0.19	0.9812	966.8 ± 7.92

(a) - chloroform/ethylacetate 1:10 ; (b) - value displayed with the standard deviation.

It can be seen that the TPC was greater in the fractions obtained from the sulphite black liquor. This fact is probably related with the chemical process applied to the lignin structure during the pulping process, which give rise to less degraded phenol structures. The AAI results for the separated extract fractions are summarized in Table 3.

**Figure 4:**
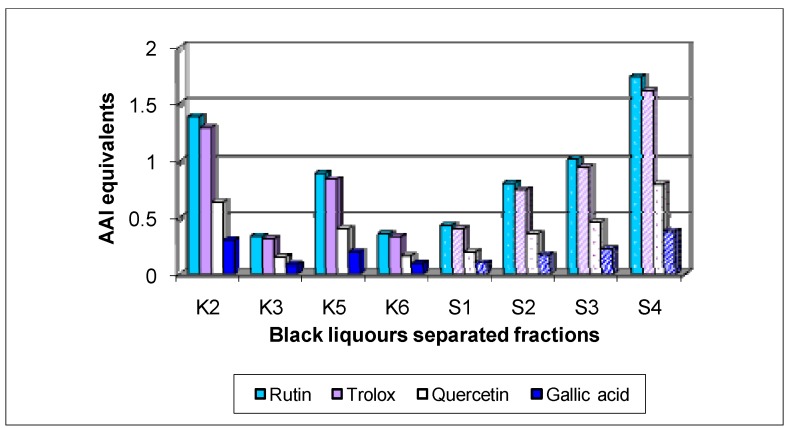
AAI equivalents of the separated fractions from kraft and sulphite black liquors in comparison with several standards.

The separated fractions from kraft black liquor showed an AAI ranging from 2.21 ± 0.27 (K3) to 9.14 ± 1.08 (K2) and the separated fractions from sulphite black liquor showed an AAI ranging from 2.85 ± 0.13 (S1) to 11.47 ± 1.65 (S4). In all cases the separated fractions presented very strong antioxidant activity, according to the scale proposed by Scherer and Godoy [22]. Figure 4 displays the AAI equivalents of the separated fractions from the kraft and sulphite black liquors, in comparison with the standards.

**Table 3:** Values of the antioxidant activity index (AAI) with different final concentrations of DPPH for the separated fractions from the kraft and sulphite black liquors.

Kraft black liquor pH = 6					Sulphite black liquor pH = 5				
Fraction	DPPH conc. (mg.mL^-1^)	Mean IC_50_	Mean AAI ^(a)^	Total Mean AAI ^(a)^	Fraction	DPPH conc. (mg.mL^-1^)	Mean IC_50_	Mean AAI ^(a)^	Total Mean AAI ^(a)^
K2	31.6	4.25	8.33 ± 0.08	9.14 ± 1.08	S1	31.6	9.92	2.68 ± 0.02	2.85 ± 0.13
	49.0	6.67	8.52 ± 0.16			49.0	17.43	2.94 ± 0.02	
	78.8	8.70	10.56 ± 0.18			78.8	27.23	2.93 ± 0.07	
K3	31.6	16.55	1.98 ± 0.02	2.21 ± 0.27	S 2	31.6	5.51	4.72 ± 0.04	5.22 ± 0.44
	49.0	25.20	2.09 ± 0.04			49.0	9.22	5.22 ± 0.02	
	78.8	32.16	2.57 ± 0.01			78.8	13.31	5.72 ± 0.09	
K5	31.6	6.14	5.30 ± 0.06	5.89 ± 0.63	S 3	31.6	4.17	5.99 ± 0.02	6.74 ± 0.62
	49.0	9.23	5.68 ± 0.02			49.0	6.72	6.83 ± 0.16	
	78.8	12.24	6.70 ± 0.07			78.8	9.88	7.38 ± 0.13	
K6	31.6	12.97	2.09 ± 0.01	2.35 ± 0.25	S 4	31.6	2.59	9.48 ± 0.19	11.47± 1.65
	49.0	17.60	2.31 ± 0.02			49.0	3.89	11.70 ± 0.21	
	78.8	25.34	2.67 ± 0.04			78.8	5.52	13.24 ± 0.33	

(a) - value displayed with the standard deviation.

As occurred with TPC, in a global appreciation the sulphite separated fractions have bigger relative AAI values, which is in accordance with a positive correlation between TPC and antioxidant activity referred by some authors [15,21].

### 2.3. Identification of compounds in the separated fractions

The fractions separated by column chromatography were submitted to mass spectrometry analysis, and the results were compared to others in the literature of natural products, mainly from eucalyptus, and the characteristic bands of functional groups were identified by ^1^H-NMR and FTIR. The presence of 17 compounds was ascertained in the isolated fractions, namely *epi*-syringaresinol, eudesmin, lariciresinol, 3-methoxy-6-(3,4,5-trimethoxyphenethyl)benzene-1,2-diol, (*Z*)-3-methoxy-6-(3,4,5-tri-methoxystyryl)benzene-1,2-diol, 1-(2,4-dihydroxyphenyl)-3-(3,4-dimethoxyphenyl)-propan-1-one, galanganal, 2’,4’,6’,4-tetrahydroxydihydrochalcone, naringenin, methyl 3,4,5-trimethoxybenzoate, syringylacetone, syringic acid, acetosyringone, 4-propenylsyringol, syringaldehyde, syringol and benzene-1,2,3-triol. K1 has proven to be sulfur in a cyclic S_8_ form; however, all other compounds obtained are phenols. K2 was the only pure phenol (syringol) obtained; all the other fractions were mixtures of phenols.

## 3. Experimental

### 3.1. General

The pulping liquors used in the investigation were supplied by two Portuguese pulp mills: Celtejo – Empresa de Celulose do Tejo, S.A. (kraft black liquor) and Caima – Indústria de Celulose S.A. (sulphite black liquor). Chloroform, ethyl acetate and methanol were from analytical grade, purchased from Sigma, 2,2-diphenyl-1-picrylhydrazyl, gallic acid, rutin, trolox and quercetin was purchased from Sigma (pure grade). All extracts and separated fractions were monitored by thin-layer chromatography (TLC) on aluminum plates precoated with Merck silica gel 60 F_254_ (0.25 mm) using chloroform/ ethyl acetate (1:10) and the spots have been examined under 254 nm UV light.

^1^H spectra were recorded in CDCl_3_ solutions on a Brücker ACP 250 (250.13 MHz) spectrometer. Chemical shifts are reported in ppm relatively to residual solvent signals or Me_4_Si and coupling constants (*J*) are given in Hz. Visible spectra (Vis) were recorded on a Perkin-Elmer Lambda 6 spectrophotometer in methanol. Wavelength of maximum absorption (l_max_) is reported in nm. Infrared spectra (IR) were performed on a FTIR Bruker IFS-28 spectrometer. Time-of-Flight Mass Spectra (TOFMS) were recorded in a Waters-Micromass GC-TOF spectrometer, operating in EI.

### 3.2. Crude extraction from kraft and sulphite black liquors

Aliquots of of kraft or sulphite black liquors (100 mL) were diluted in distilled water (900 mL). A volume from each solution (200 mL) was treated with 4N HCl to obtain solutions with a pH of 1, 2, 3, 4 and 5 for sulphite black liquor and 1, 2, 4, 6 and 12 for kraft black liquor. These solutions were extracted with ethyl acetate (600 mL) in a single extraction step [20]. Ethyl acetate was removed by vacuum evaporation and reutilized. The crude extracts were dried overnight under vacuum in a dessicator.

### 3.3.Separation of fractions from crude extracts

#### 3.3.1. Separation of fractions from crude extract of kraft black liquor

A volume of 4N HCl (100 m) was added under stirring to kraft black liquor (1 L) gaving rise to a solution of pH = 6. This solution was centrifuged at 4,500 rpm for 7 min; the liquid phase was decanted and extracted with ethyl acetate using a solution-ethyl acetate volume ratio of 1:3 (v/v) in a single extraction step. The ethyl acetate was removed by vacuum evaporation and the crude extract (2.213 g) was subjected to column chromatography (CC) using chloroform/ethyl acetate 1:10 as eluent.

#### 3.3.2. Separation of fractions from crude extract of sulphite black liquor

Sulphite black liquor (500 mL) was dissolved in distilled water (1 L), this solution is extracted with ethyl acetate using a solution-ethyl acetate volume ratio of 1:3 (v/v) in a single extraction stage. The ethyl acetate was removed by vacuum evaporation and the crude extract (12.791 g) was subjected to column chromatography (CC) using chloroform/ethyl acetate 1:10 as eluent.

#### 3.3.3 Characterization of the fractions

**K1** – TOFMS (g·mol^-1^): 255.78 g·mol^-1^.

**K2** – ^1^H-NMR (CDCl_3_) δ (ppm): 6.77 (d, *J* = 8.6 Hz, 1H, Ar-*H*), 6.56 (d, *J* = 8.6 Hz, 2H, Ar-*H*), 5.53 (s, 1H), 3.85 (s, 6H). IR u (cm^-1^): 3433 (s, O-H), 1215 (s, C_ar_-OH), 1032 (m, C_ar_-O-C). TOFMS (g·mol^‑1^): 154.06.

**K3** – ^1^H-NMR (CDCl_3_) δ (ppm): 9.81 (s, C-*H* aldehyde), 7.56–7.49 (m), 7.45–7.37 (m), 7.02 (d, *J* = 8.5 Hz), 6.90 (dd, *J* = 11.9, 8.1 Hz), 6.71 (s), 6.40 (s), 6.13 (d, *J* = 31.3 Hz), 5.72–5.43 (m), 4.13–3.77 (m), 2.54 (s), 1.23 (s), 1.08–0.74 (m). IR u (cm^-1^): 3502 (s, O-H), 3415 (s, O-H), 2841 (w, C-H aldehyde), 1669 (s, C=O), 1607 (s, C=O), 1213 (s, C_ar_-OH), 1031 (m, C_ar_-O-C). TOFMS (g·mol^-1^): 334.14; 332.13; 302.12; 272.07; 210.09; 196.07; 182.07.

**K4** – ^1^H NMR (CDCl_3_) δ (ppm): 9.74 (s, C-*H* aldehyde), 7.19 (s), 7.09 (s), 6.82 (s), 6.68 (s), 6.36 (d, *J* = 8.9 Hz), 3.87 (dt, *J* = 11.3, 5.6 Hz), 3.78 (s), 2.52 (d, *J* = 0.8 Hz). IR u (cm^-1^): 3504 (s, O-H), 3372 (s, O-H), 3296 (s, O-H), 2841 (w, C-H aldehyde), 1666 (s, C=O), 1249 (m, C_ar_-OH), 1207 (s, C_ar_-OH), 1114 (s), 1031 (m, C_ar_-O-C). TOFMS (g·mol^‑1^): 196.07; 182.07.

**K5** – ^1^H NMR (CDCl_3_) δ (ppm): 7.24 (s), 6.82 (s), 6.57 (t, *J* = 3.3 Hz), 6.57 (t, *J* = 3.3 Hz), 5.48 (d, *J* = 5.0 Hz), 5.48 (d, *J* = 5.0 Hz), 4.86–4.81 (m), 4.72 (s), 4.72 (s), 4.41 (s), 4.34 (d, *J* = 35.7 Hz), 4.27 (s), 4.24–4.14 (m), 4.10 (s), 4.04–3.87 (m,), 4.24–3.71 (m), 3.86 (s), 3.79 (s), 3.49–3.43 (m), 3.31 (d, *J* = 2.8 Hz), 3.31 (d, *J* = 2.8 Hz), 3.08 (s), 3.08 (s), 1.23 (s), 1.23 (s). IR u (cm^-1^): 3421 (s, O-H), 2841 (w, C-H aldehyde), 1651 (m, C=O), 1611 (s, C=O), 1275 (m, C_ar_-OH), 1214 (s, C_ar_-OH), 1155 (m, C_ar_-OH), 1033 (m, C_ar_-O-C). TOFMS (g·mol^-1^): 418.16; 332.13; 196.07; 194.09; 126.03.

**K6 – **^1^H NMR (CDCl_3_) δ (ppm): 8.69 (s, C-*H* aldehyde) 7.80 (s), 7.24 (s), 7.19 (s), 6.83–6.77 (m), 6.56 (d, *J* = 6.5 Hz), 6.46–6.35 (m), 4.70 (d, *J* = 4.2 Hz), 4.30–4.21 (m), 3.86 (ddd, *J* = 8.9, 5.5, 2.1 Hz), 3.43 (s), 3.11–3.03 (m), 2.75 (t, *J* = 6.6 Hz), 2.63–2.54 (m), 1.97 (d, *J* = 4.3 Hz), 1.44 (d, *J* = 6.4 Hz), 1.22 (s), 0.89–0.77 (m). IR u (cm-1): 3428 (s, O-H), 2842 (w, C-H aldehyde), 1610 (s, C=O), 1213 (s, C_ar_-OH), 1154 (m, C_ar_-OH), 1036 (m, C_ar_-O-C). TOFMS (g·mol^-1^): 418.16; 360.16; 280.10; 182.07.

**S1 – **^1^H NMR (CDCl_3_) δ (ppm): 9.63 (s, C-*H* aldehyde), 7.67 (dt, *J* = 1.5, 0.7 Hz), 7.23 (dd, *J* = 3.6, 0.8 Hz), 6.58 (ddd, *J* = 3.6, 1.6, 0.8 Hz), 6.53 (s), 6.38 (s), 5.39 (s), 5.15–4.94 (m), 3.84 (t, *J* = 2.6 Hz), 3.28 (d, *J* = 6.7 Hz), 2.42 (d, *J* = 16.3 Hz), 1.83 (dd, *J* = 6.4, 1.4 Hz), 1.22 (s). IR u (cm-1): 3434 (s, O-H), 2840 (w, C-H aldehyde), 1762 (m, C=O), 1717 (m, C=O), 1677 (m, C=O), 1652 (m, C=O), 1613 (s, C=O), 1241 (s, C_ar_-OH), 1215 (s, C_ar_-OH), 1038 (m, C_ar_-O-C). TOFMS (g·mol^‑1^): 210.09; 182.07.

**S2 – **^1^H NMR (CDCl_3_) δ (ppm): 9.61 (s, C-*H* aldehyde), 7.72–7.59 (m), 7.24–7.20 (m), 7.28–7.10 (m), 6.77–6.43 (m), 6.42–6.33 (m), 6.83–5.78 (m), 6.26–6.14 (m), 6.12–5.74 (m), 5.59–5.47 (m), 5.64–5.37 (m), 5.48–5.36 (m), 5.11–4.93 (m), 5.18–4.95 (m), 4.03–3.59 (m), 3.93–3.54 (m), 3.53 (s), 3.34–3.19 (m), 3.36–3.14 (m), 2.51–2.33 (m), 2.48–2.26 (m), 2.12–1.97 (m), 1.92–1.73 (m), 1.93–1.75 (m), 1.48–1.12 (m), 1.18 (dd, *J* = 13.3, 4.3 Hz), 1.07–0.80 (m). IR u (cm-1): 3430 (s, O-H), 2849 (m, C-H aldehyde),, 1732 (m, C=O), 1678 (m, C=O), 1243 (s, C_ar_-OH), 1216 (s, C_ar_-OH), 1113 (s, C_ar_-OH), 1039 (m, C_ar_-O-C). TOFMS (g·mol^-1^): 210.09; 196.07; 182.07.

**S3 – **^1^H NMR (CDCl_3_) δ (ppm): 9.79 (s, C-*H* aldehyde), 7.31 (s), 7.13 (s), 6.86 (s), 6.71 (s), 6.39 (s), 6.30 (s), 5.57 (s), 3.99–3.82 (m), 3.74 (s), 3.55 (d, *J* = 16.8 Hz), 2.50 (s), 2.13 (s), 2.03 (d, *J* = 5.4 Hz), 1.23 (t, *J* = 7.2 Hz), 0.85 (d, *J* = 7.5 Hz). IR u (cm-1): 3504 (s, O-H), 3425 (s, O-H), 1706 (m, C=O), 1657 (m, C=O), 1611 (s, C=O), 1263 (m, C_ar_-OH), 1211 (s, C_ar_-OH), 1163 (m, C_ar_-OH), 1110 (s), 1032 (m, C_ar_-O-C). TOFMS (g·mol^‑1^): 386.17; 332.13; 302.12; 210.09.

**S4 – **^1^H NMR (CDCl_3_) δ (ppm): 12.44 (d, *J* = 0.7 Hz, CO_2_*H*), 9.79 (s, C-*H* aldehyde), 9.63 (s, C-*H* aldehyde), 7.70 (d, *J* = 1.4 Hz), 7.68–7.65 (m, 4), 7.64–7.61 (m), 7.58 (dd, *J* = 1.9, 0.9 Hz), 7.55 (dd, *J* = 2.1, 1.2 Hz), 7.49–7.46 (m), 7.24 (s), 7.02–6.80 (m), 6.70 (s), 6.65–6.60 (m), 6.58–6.52 (m), 6.40 (d, *J* = 11.1 Hz), 6.25 (d, *J* = 3.2 Hz), 5.93–5.86 (m), 3.99–3.73 (m), 2.60 (s), 2.53–2.41 (m), 2.21 (d, *J* = 0.8 Hz), 1.22 (s), 0.89 (s). IR u (cm^-1^): 3428 (s, O-H), 2845 (m, C-H aldehyde), 1751 (w, C=O), 1704 (m, C=O), 1615 (s, C=O), 1276 (m, C_ar_-OH), 1216 (s, C_ar_-OH), 1156 (s, C_ar_-OH), 1037 (m, C_ar_-O-C). TOFMS (g·mol^-1^): 418.16; 274.09; 226.09; 210.09; 198.05; 182.07.

#### 3.3.4. Identification of compounds in the fractions

Table 4 displays the proposed structures for the compounds present in the isolated fractions. 

**Table 4:** Compounds identified by mass spectroscopy and comparison with literature.

Possible structure/name	Formula	Molecular weight	Exact mass found	Reference	Fractions
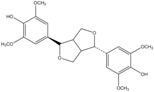	C_22_H_26_O_8_	418.44	418.16	[24]	**K5; K6; S4**
Epi-syringaresinol					
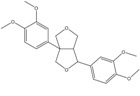	C_22_H_26_O_6_	386.44	386.17	[25]	**S3**
Eudesmin					
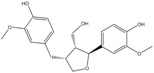	C_20_H_24_O_6_	360.40	360.16	[26]	**K6**
Lariciresinol					
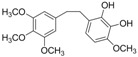	C_18_H_22_O_6_	334.36	334.14	[27]	**K3**
3-Methoxy-6-(3,4,5-trimethoxyphenethyl)benzene-1,2-diol					
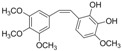	C_18_H_20_O_6_	332.35	332.13	[27,28]	**K3; K5; S3**
(*Z*)-3-Methoxy-6-(3,4,5-trimethoxystyryl)benzene-1,2-diol					
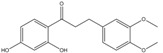	C_17_H_18_O_5_	302.32	302.12	[29,30]	**K3; S3**
1-(2,4-Dihydroxyphenyl)-3-(3,4-dimethoxyphenyl)-propan-1-one					
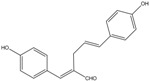	C_18_H_16_O_3_	280.32	280.10	[31]	**K6; S5**
Galanganal					
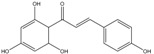	C_15_H_14_O_5_	274.27	274.09	[32]	**S4**
2',4’,6′,4-Tetrahydroxydihydrochalcone					
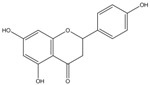	C_15_H_12_O_5_	272.25	272.07	[33]	**K3**
Naringenin					
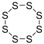	S_8_	256.52	255.78		**K1**
Sulphur					
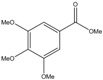	C_11_H_14_O_5_	226.23	226.09	[34]	**S4**
Methyl 3,4,5-trimethoxybenzoate					
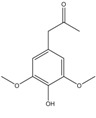	C_11_H_14_O_4_	210.23	210.09	[35]	**K3; S1; S2; S3; S4**
Syringylacetone					
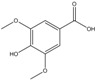	C_9_H_10_O_5_	198.17	198.05	[36]	**S4**
Syringic acid					
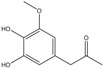	C_10_H_12_O_4_	196.20	196.07	[35]	**K3; K4; K5; S2**
Acetosyringone					
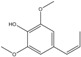	C_11_H_14_O_3_	194.23	194.09	[35]	**K5**
4-Propenylsyringol					
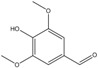	C_9_H_10_O_4_	182.17	182.07	[35,37]	**K3; K4; K6; S1; S2; S4**
Syringaldehyde					
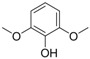	C_8_H_10_O_3_	154.16	154.06	[35,38]	**K2**
Syringol					
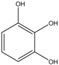	C_6_H_6_O_3_	126.11	126.03	[37]	**K5**
Benzene-1,2,3-triol					

### 3.4. Determination of Total Phenolic Content (TPC)

Total phenolic content was estimated by the Folin–Ciocalteu colorimetric method, based on the procedure of Bonoli *et al*. [21], using gallic acid as a standard phenolic compound. Briefly, a methanolic solution from each extract or separated fraction (50 μL) was diluted in water (450 μL) and 0.2 N Folin-Ciocalteu reagent solution (2.50 mL) was added and the mixture was shaken for 5 min. After this procedure, Na_2_CO_3_ (75 g·L^-1^, 2 mL) was added and the mixture was shaken once again for 1.5 h at 30 ºC. The absorbance at 765 nm (25 ºC) was evaluated using glass cuvettes. A linear calibration curve of gallic acid, in the range 100–2,000 mg·L^-1 ^was prepared (*A* = 0.000890*c*, *r*^2^ = 0.998). The total phenolic content was expressed as gallic acid equivalents (GAE) in milligrams per gram of dry material.

### 3.5. Antioxidant activity

The antioxidant activity of the samples and standards was determined by the radical scavenging activity method using 2,2-diphenyl-1-picrylhydrazyl radical (DPPH) as described by Scherer and Godoy [22]. The concentration of DPPH was assessed by plotting the DPPH calibration curve from 4.28 to 85.6 μg·mL^-1^ (*A* = 0.0250*c*, *r*^2^ = 0.999). Each aliquot of methanolic solutions of the samples or standards (0.1 mL) at different concentrations was added to a methanolic solution of DPPH (3.9 mL). Three DPPH solutions were tested: 78.8, 49.0 and 31.6 μg·mL^-1^. The blank sample consisted of methanol (0.1 mL) added to DPPH solution (3.9 mL). The tests were carried out in triplicate. After a 90 min incubation period at room temperature in the dark, the absorbance was measured at 517 nm. The radical scavenging activity was calculated as follows: I% = [(Abs_0_-Abs_1_)/Abs_0_] × 100, where Abs_0_ was the absorbance of the blank and Abs_1_ was the absorbance in the presence of the test compound at different concentrations. The IC_50_ (concentration providing 50% inhibition) was calculated graphically using a calibration curve in the linear range by plotting the extract concentration *vs.* the corresponding scavenging effect. The antioxidant activity was expressed as the antioxidant activity index (AAI), calculated as follows:

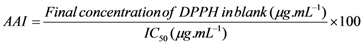
(1)


This method was chosen because it provides a constant value, independent of the DPPH concentration and sample used.

## 4. Conclusions

The investigations into scavenging effects of crude extracts and separated fractions from kraft and sulphite black liquors on the DPPH radical have revealed that they can be effective and consequentely can provide a source of antioxidants with very strong antioxidant effects, particularly in the case of sulphite liquor. The separation of fractions by column chromatography can eliminate potential hazardous compounds, like S_8_ in K1, and supply phenolic fractions with very high AAI. Despite the fact that mixtures of compounds are found in most fractions, this procedure was revealed to allow a recovery of several phenolic compounds from the black liquors which can be an important and low cost source of natural antioxidants. Nevertheless, these highly valued antioxidant compounds need safety testing, since their natural origin does not assure their safe behavior. 
